# Crystal structure of 11-(2,3-di­meth­oxy­phen­yl)-14-methyl-12-oxa-8,14-di­aza­tetra­cyclo­[8.3.3.0^1,10^.0^2,7^]hexa­deca-2(7),3,5-triene-9,13-dione

**DOI:** 10.1107/S2056989015006386

**Published:** 2015-04-09

**Authors:** M. P. Savithri, M. Suresh, R. Raghunathan, R. Raja, A. SubbiahPandi

**Affiliations:** a Department of Physics, Queen Mary’s College (Autonomous), Chennai 600 004, India; b Department of Organic Chemistry, University of Madras, Guindy Campus, Chennai 600 025, India; c Department of Physics, Presidency College (Autonomous), Chennai 600 005, India

**Keywords:** crystal structure, di­aza­tetra­cyclhexa­deca­trienedione, N—H⋯O hydrgen bonds

## Abstract

The title compound, C_22_H_22_N_2_O_5_, contains two conformationally similar mol­ecules (*A* and *B*) in its the asymmetric unit (r.m.s. overlay fit for the 29 non-H atoms = 0.194 Å). In each mol­ecule, the lactone ring has an envelope conformation with the spiro C atom as the flap. In the crystal, *A*+*A* and *B*+*B* inversion dimers linked by pairs of N—H⋯O hydrgen bonds occur; in both cases, *R*_2_^2^(8) loops are generated. A weak C—H⋯O inter­action is also observed, which links the dimers into [010] chains.

## Related literature   

For general background and the biological and pharmacological properties of quinoline derivatives, see: Michael (1997 ▸). For a related structure, see: Vennila *et al.* (2011 ▸).
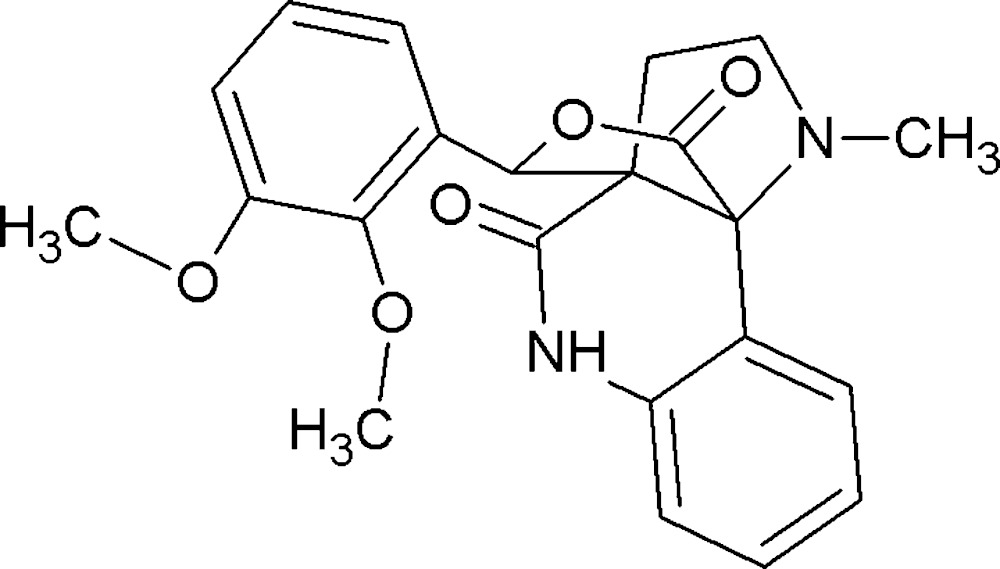


## Experimental   

### Crystal data  

C_22_H_22_N_2_O_5_
*M* *_r_* = 394.42
Triclinic, 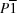

*a* = 10.1360 (4) Å
*b* = 10.3198 (4) Å
*c* = 18.8973 (7) Å
α = 89.079 (2)°
β = 74.955 (2)°
γ = 89.406 (2)°
*V* = 1908.64 (13) Å^3^
*Z* = 4
Mo *K*α radiation
μ = 0.10 mm^−1^
*T* = 293 K
0.35 × 0.30 × 0.30 mm

### Data collection  

Bruker Kappa APEXII CCD diffractometer
Absorption correction: multi-scan (*SADABS*; Bruker, 2004 ▸) *T*_min_ = 0.967, *T*_max_ = 0.971
35145 measured reflections
6717 independent reflections
5408 reflections with *I* > 2σ(*I*)
*R*_int_ = 0.028

### Refinement  

*R*[*F* ^2^ > 2σ(*F* ^2^)] = 0.047
*wR*(*F* ^2^) = 0.128
*S* = 1.02
6717 reflections
532 parameters
H atoms treated by a mixture of independent and constrained refinement
Δρ_max_ = 0.65 e Å^−3^
Δρ_min_ = −0.54 e Å^−3^

### 

Data collection: *APEX2* (Bruker, 2004 ▸); cell refinement: *SAINT* (Bruker, 2004 ▸); data reduction: *SAINT*; program(s) used to solve structure: *SHELXS97* (Sheldrick, 2008 ▸); program(s) used to refine structure: *SHELXL97* (Sheldrick, 2008 ▸); molecular graphics: *ORTEP-3 for Windows* (Farrugia, 2012 ▸); software used to prepare material for publication: *SHELXL97* and *PLATON* (Spek, 2009 ▸).

## Supplementary Material

Crystal structure: contains datablock(s) global, I. DOI: 10.1107/S2056989015006386/hb7391sup1.cif

Structure factors: contains datablock(s) I. DOI: 10.1107/S2056989015006386/hb7391Isup2.hkl

Supporting information file. DOI: 10.1107/S2056989015006386/hb7391Isup3.cml

**Fig. 1.**
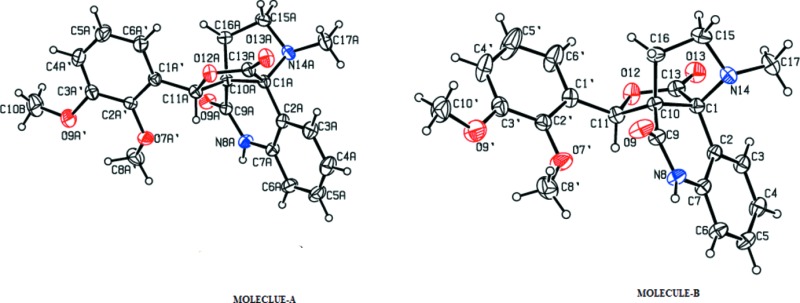
The molecular structure of the title compound with the atom-numbering scheme. Displacement ellipsoids are drawn at the 30% probability level. H atoms are omitted for clarity.

**Fig. 2.**
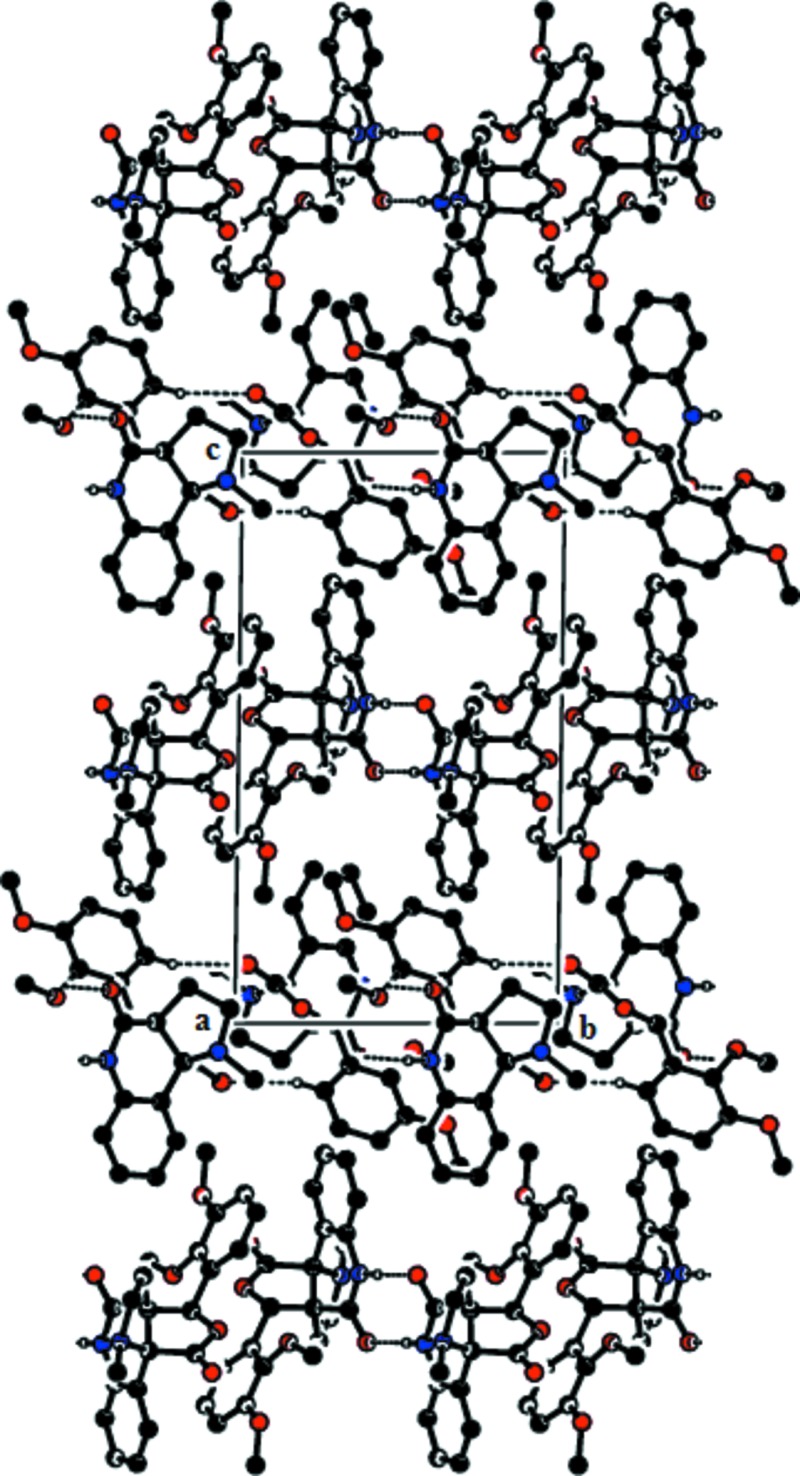
The molecular packing is viewed along the a axis. Dashed lines show the intermolecular N—H···O and C—H···O hydrogen bonds. H atoms not involved in hydrogen bonding have been omitted for clarity.

. DOI: 10.1107/S2056989015006386/hb7391fig1.tif
The mol­ecular structure of the title compound with the atom-numbering scheme. Displacement ellipsoids are drawn at the 30% probability level. H atoms are omitted for clarity.

a . DOI: 10.1107/S2056989015006386/hb7391fig2.tif
The mol­ecular packing is viewed along the *a* axis. Dashed lines show the inter­molecular N—H⋯O and C—H⋯O hydrogen bonds. H atoms not involved in hydrogen bonding have been omitted for clarity.

**Fig. 3.**
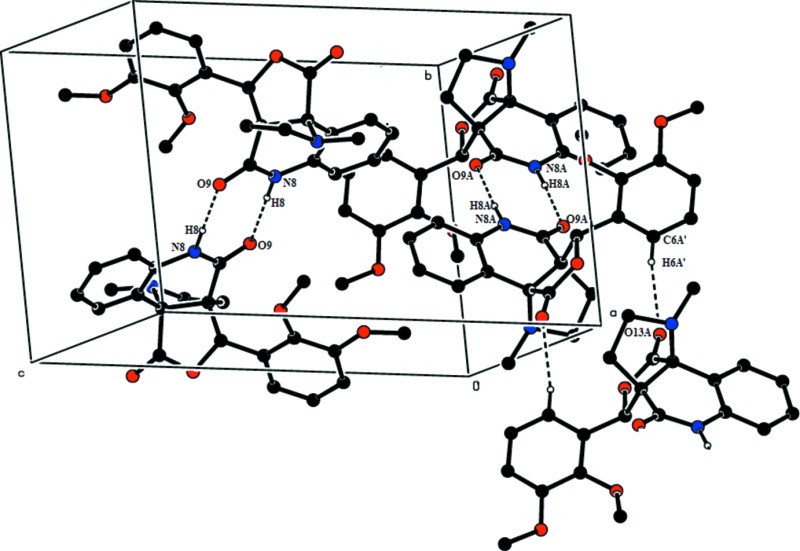
A partial view of the N8—H8···O9, N8A—H8A···O9A and C6A'—H6A'···O13A hydrogen-bonding interactions along the c axis.

A A A A A A c . DOI: 10.1107/S2056989015006386/hb7391fig3.tif
A partial view of the N8—H8⋯O9, N8*A*—H8*A*⋯O9*A* and C6*A*′—H6*A*′⋯O13*A* hydrogen-bonding inter­actions along the *c* axis.

CCDC reference: 1056691

Additional supporting information:  crystallographic information; 3D view; checkCIF report

## Figures and Tables

**Table 1:** Hydrogen-bond geometry (, )

*D*H*A*	*D*H	H*A*	*D* *A*	*D*H*A*
N8H8O9^i^	0.89(3)	2.01(3)	2.903(2)	177(2)
N8*A*H8*A*O9*A* ^ii^	0.89(2)	2.07(3)	2.958(2)	175(2)
C6*A*H6*A*O13*A* ^iii^	0.93	2.43	3.322(3)	161

Symmetry codes: (i) 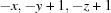; (ii) 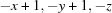; (iii) 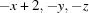.

## supplementary crystallographic information

### S1. Comment

Quinolines exhibit physico-chemical activities which are useful in the field of pharmaceuticals and agrochemicals. Their derivatives are also present in a wide variety of natural products involved in several biological activities (Michael, 2006). The crystal structure of the title compound is presented here as a part of our on-going structural studies on quinoline derivatives.

The molecular structure of molecule (A) and molecule (B) is shown in Fig.1. The furan ring (O12A/C11A-C10A/C1A/C13A) of (A) exhibits an envelope conformation with C10A as the flap atom. The furan ring (O12/C11-C10/C1/C13) of (B) exhibits an envelope conformation with C10 as the flap atom. The quinoline ring (N8A/C1A-C10A) is almost coplanar showing a dihedral angle of 2.9 (8)° with the pyridine ring (N8A/C7A/C2A-C1A/C9A-C10A) of molecule (A) and is perpendicular with the pyridine ring (N8/C7/C2-C1/C9-C10) of molecule (B) inclined at an angle of 88.3 (8)°. The sum of angles at N8, N8A of the quinoline rings (360°) is in accordance with sp^2^ hybridization.

In the crystal of two independent molecules, hydrogen-bonded chains running along bc plane are generated by connecting neighbouring molecules via N-H···O, C-H···O hydrogen bonds forming a two dimensional structure (Fig.2.) The hydrogen bonds of N8-H8···O9 and N8A-H8A···O9A forming inversion dimers but enclosing smaller R^2^_2_(8) loops and the hydrogen bond of C6A'-H6A'···O13A forms a one dimensional chain along [010] as shown in Fig.3.

### S2. Experimental

A mixture of methyl 2-(hydroxy(m-tolyl)methyl)acrylate (252mgs, 1 mmol), isatin (161.7mgs, 1.1 mmol) and sarcosine (97.9mgs, 1.1 mmol) was placed in a round bottom flask and melted at 180°C until completion of the reaction was evidenced by TLC analysis. After completion of the reaction, the crude product was washed with 5ml of ethylacetate and hexane mixture (1:4 ratio) which successfully provided the pure product as colorless solid. The product was dissolved in ethyl acetate and heated for two minutes. The resulting solution was subjected to crystallization by slow evaporation of the solvent for 48 hours resulting in the formation of colourless blocks.

### S3. Refinement

All H atoms were fixed geometrically and allowed to ride on their parent C atoms, with C-H distances fixed in the range 0.93-0.98 Å with *U*_iso_(H) = 1.5*U*_eq_(C) for methyl H 1.2*U*_eq_(C) for other H atoms.

### Crystal data

**Table:**

C_22_H_22_N_2_O_5_	*Z* = 4
*M**_r_* = 394.42	*F*(000) = 832
Triclinic, *P*1	*D*_x_ = 1.373 Mg m^−^^3^
Hall symbol: -P 1	Mo *K*α radiation, λ = 0.71073 Å
*a* = 10.1360 (4) Å	Cell parameters from 6717 reflections
*b* = 10.3198 (4) Å	θ = 1.1–25.0°
*c* = 18.8973 (7) Å	µ = 0.10 mm^−^^1^
α = 89.079 (2)°	*T* = 293 K
β = 74.955 (2)°	Block, colourless
γ = 89.406 (2)°	0.35 × 0.30 × 0.30 mm
*V* = 1908.64 (13) Å^3^	

### Data collection

**Table:**

Bruker Kappa APEXII CCD diffractometer	6717 independent reflections
Radiation source: fine-focus sealed tube	5408 reflections with *I* > 2σ(*I*)
Graphite monochromator	*R*_int_ = 0.028
ω and φ scans	θ_max_ = 25.0°, θ_min_ = 1.1°
Absorption correction: multi-scan (*SADABS*; Bruker, 2004)	*h* = −12→12
*T*_min_ = 0.967, *T*_max_ = 0.971	*k* = −12→12
35145 measured reflections	*l* = −22→22

### Refinement

**Table:**

Refinement on *F*^2^	Secondary atom site location: difference Fourier map
Least-squares matrix: full	Hydrogen site location: inferred from neighbouring sites
*R*[*F*^2^ > 2σ(*F*^2^)] = 0.047	H atoms treated by a mixture of independent and constrained refinement
*wR*(*F*^2^) = 0.128	*w* = 1/[σ^2^(*F*_o_^2^) + (0.0555*P*)^2^ + 1.1105*P*] where *P* = (*F*_o_^2^ + 2*F*_c_^2^)/3
*S* = 1.02	(Δ/σ)_max_ < 0.001
6717 reflections	Δρ_max_ = 0.65 e Å^−^^3^
532 parameters	Δρ_min_ = −0.54 e Å^−^^3^
0 restraints	Extinction correction: *SHELXL97* (Sheldrick, 2008), Fc^*^=kFc[1+0.001xFc^2^λ^3^/sin(2θ)]^-1/4^
Primary atom site location: structure-invariant direct methods	Extinction coefficient: 0.0073 (9)

### Special details

**Table:**

Geometry. All esds (except the esd in the dihedral angle between two l.s. planes) are estimated using the full covariance matrix. The cell esds are taken into account individually in the estimation of esds in distances, angles and torsion angles; correlations between esds in cell parameters are only used when they are defined by crystal symmetry. An approximate (isotropic) treatment of cell esds is used for estimating esds involving l.s. planes.
Refinement. Refinement of F^2^ against ALL reflections. The weighted R-factor wR and goodness of fit S are based on F^2^, conventional R-factors R are based on F, with F set to zero for negative F^2^. The threshold expression of F^2^ > 2sigma(F^2^) is used only for calculating R-factors(gt) etc. and is not relevant to the choice of reflections for refinement. R-factors based on F^2^ are statistically about twice as large as those based on F, and R- factors based on ALL data will be even larger.

### Fractional atomic coordinates and isotropic or equivalent isotropic displacement parameters (Å^2^)

**Table:**

	*x*	*y*	*z*	*U*_iso_*/*U*_eq_	
C10B	1.0615 (3)	0.7030 (3)	−0.25020 (16)	0.0810 (9)	
H10A	1.0438	0.7943	−0.2528	0.121*	
H10B	1.0127	0.6574	−0.2792	0.121*	
H10C	1.1577	0.6868	−0.2686	0.121*	
H8A	0.517 (2)	0.461 (2)	0.0654 (12)	0.049 (6)*	
H8	0.057 (3)	0.546 (2)	0.4402 (13)	0.061 (7)*	
C1A	0.71903 (18)	0.15796 (17)	0.06757 (10)	0.0323 (4)	
O12A	0.95534 (13)	0.20658 (13)	0.02653 (8)	0.0433 (4)	
O9A	0.61164 (15)	0.38520 (14)	−0.05792 (7)	0.0448 (4)	
N8A	0.57191 (17)	0.39272 (17)	0.06429 (9)	0.0379 (4)	
C9A	0.63693 (18)	0.34543 (18)	−0.00142 (10)	0.0324 (4)	
O13A	0.90527 (15)	0.03882 (14)	0.10304 (9)	0.0530 (4)	
O7A'	0.86006 (16)	0.55593 (14)	−0.04580 (8)	0.0513 (4)	
C11A	0.88282 (18)	0.30883 (18)	−0.00169 (10)	0.0337 (4)	
H11A	0.8642	0.3801	0.0333	0.040*	
C7A	0.5767 (2)	0.34058 (19)	0.13261 (10)	0.0367 (4)	
C10A	0.74578 (18)	0.24380 (17)	−0.00209 (10)	0.0301 (4)	
N14A	0.64191 (16)	0.04987 (15)	0.04930 (9)	0.0389 (4)	
C1A'	0.97041 (19)	0.35816 (18)	−0.07344 (11)	0.0363 (4)	
C2A	0.64674 (19)	0.22556 (19)	0.13708 (10)	0.0355 (4)	
C16A	0.7560 (2)	0.14502 (19)	−0.06377 (11)	0.0400 (5)	
H16A	0.6961	0.1700	−0.0943	0.048*	
H16B	0.8489	0.1397	−0.0943	0.048*	
C13A	0.8671 (2)	0.12459 (19)	0.06982 (11)	0.0378 (5)	
C2A'	0.9520 (2)	0.48460 (18)	−0.09543 (11)	0.0362 (4)	
C3A'	1.0346 (2)	0.5333 (2)	−0.16135 (12)	0.0457 (5)	
C3A	0.6440 (2)	0.1761 (2)	0.20631 (12)	0.0504 (6)	
H3A	0.6921	0.1004	0.2105	0.060*	
C15A	0.7124 (2)	0.0159 (2)	−0.02557 (12)	0.0449 (5)	
H15A	0.6518	−0.0293	−0.0489	0.054*	
H15B	0.7912	−0.0388	−0.0265	0.054*	
C6A	0.5070 (2)	0.4038 (2)	0.19557 (12)	0.0512 (6)	
H6A	0.4620	0.4818	0.1921	0.061*	
O9A'	1.0186 (2)	0.66034 (16)	−0.17763 (10)	0.0789 (6)	
C6A'	1.0697 (2)	0.2811 (2)	−0.11824 (14)	0.0544 (6)	
H6A'	1.0829	0.1963	−0.1039	0.065*	
C4A'	1.1314 (3)	0.4546 (2)	−0.20506 (14)	0.0599 (6)	
H4A'	1.1855	0.4864	−0.2493	0.072*	
C4A	0.5714 (3)	0.2371 (3)	0.26883 (13)	0.0615 (7)	
H4A	0.5680	0.2010	0.3147	0.074*	
C17A	0.6138 (2)	−0.0621 (2)	0.09891 (14)	0.0548 (6)	
H17A	0.5677	−0.0341	0.1473	0.082*	
H17B	0.6983	−0.1040	0.1000	0.082*	
H17C	0.5571	−0.1219	0.0821	0.082*	
C5A	0.5043 (3)	0.3513 (3)	0.26324 (13)	0.0610 (7)	
H5A	0.4568	0.3934	0.3054	0.073*	
C8A'	0.7778 (3)	0.6521 (2)	−0.06863 (16)	0.0621 (7)	
H8A1	0.7195	0.6925	−0.0264	0.093*	
H8A2	0.7228	0.6124	−0.0966	0.093*	
H8A3	0.8354	0.7163	−0.0984	0.093*	
C5A'	1.1486 (3)	0.3291 (3)	−0.18348 (15)	0.0672 (7)	
H5A'	1.2143	0.2764	−0.2133	0.081*	
N8	0.12270 (18)	0.60382 (17)	0.43819 (9)	0.0413 (4)	
O12	0.29646 (15)	0.96066 (13)	0.46254 (8)	0.0480 (4)	
C1	0.36492 (18)	0.74700 (18)	0.42665 (10)	0.0334 (4)	
C2	0.31784 (19)	0.71338 (18)	0.35954 (10)	0.0348 (4)	
C7	0.1971 (2)	0.64514 (18)	0.36828 (10)	0.0360 (4)	
O9	0.09671 (17)	0.57648 (16)	0.55871 (8)	0.0579 (5)	
O13	0.50407 (15)	0.94355 (15)	0.38830 (9)	0.0551 (4)	
C10	0.25101 (19)	0.74324 (18)	0.49834 (10)	0.0322 (4)	
C11	0.1820 (2)	0.87718 (18)	0.49699 (11)	0.0374 (4)	
H11	0.1197	0.8733	0.4652	0.045*	
N14	0.46780 (16)	0.65966 (17)	0.44204 (10)	0.0423 (4)	
C9	0.1510 (2)	0.63346 (19)	0.50162 (10)	0.0372 (4)	
C13	0.4009 (2)	0.89217 (19)	0.42184 (11)	0.0388 (5)	
C1'	0.1075 (2)	0.93454 (19)	0.56880 (12)	0.0426 (5)	
C3	0.3898 (2)	0.7494 (2)	0.28913 (11)	0.0426 (5)	
H3	0.4697	0.7969	0.2823	0.051*	
C4	0.3449 (2)	0.7160 (2)	0.22934 (11)	0.0471 (5)	
H4	0.3957	0.7387	0.1824	0.057*	
C2'	−0.0258 (2)	0.8978 (2)	0.60066 (11)	0.0432 (5)	
C16	0.3306 (2)	0.7269 (2)	0.55711 (11)	0.0437 (5)	
H16C	0.3282	0.8066	0.5840	0.052*	
H16D	0.2918	0.6580	0.5914	0.052*	
C6	0.1502 (2)	0.6138 (2)	0.30803 (11)	0.0461 (5)	
H6	0.0687	0.5691	0.3145	0.055*	
C5	0.2246 (2)	0.6489 (2)	0.23883 (11)	0.0491 (5)	
H5	0.1937	0.6273	0.1983	0.059*	
O7'	−0.07771 (17)	0.8186 (2)	0.55922 (10)	0.0873 (7)	
C15	0.4763 (2)	0.6935 (2)	0.51562 (12)	0.0511 (6)	
H15C	0.5360	0.7671	0.5137	0.061*	
H15D	0.5109	0.6209	0.5389	0.061*	
C3'	−0.0965 (2)	0.9489 (2)	0.66811 (13)	0.0518 (6)	
C6'	0.1686 (3)	1.0237 (3)	0.60371 (16)	0.0825 (10)	
H6'	0.2582	1.0491	0.5828	0.099*	
O9'	−0.22558 (19)	0.9071 (3)	0.69852 (11)	0.0991 (8)	
C17	0.5998 (2)	0.6501 (2)	0.38864 (14)	0.0579 (6)	
H17D	0.6568	0.5892	0.4061	0.087*	
H17E	0.6426	0.7335	0.3817	0.087*	
H17F	0.5871	0.6212	0.3428	0.087*	
C5'	0.0973 (4)	1.0747 (3)	0.66906 (19)	0.1108 (15)	
H5'	0.1388	1.1352	0.6920	0.133*	
C4'	−0.0340 (3)	1.0379 (3)	0.70101 (16)	0.0753 (9)	
H4'	−0.0811	1.0736	0.7454	0.090*	
C8'	−0.1935 (3)	0.7448 (3)	0.58216 (18)	0.0735 (8)	
H8'1	−0.2080	0.6977	0.5415	0.110*	
H8'2	−0.2707	0.8003	0.6013	0.110*	
H8'3	−0.1827	0.6849	0.6197	0.110*	
C10'	−0.2794 (3)	0.9163 (3)	0.77410 (16)	0.0827 (9)	
H10D	−0.3707	0.8829	0.7874	0.124*	
H10E	−0.2809	1.0055	0.7881	0.124*	
H10F	−0.2236	0.8670	0.7988	0.124*	

### Atomic displacement parameters (Å^2^)

**Table:**

	*U*^11^	*U*^22^	*U*^33^	*U*^12^	*U*^13^	*U*^23^
C10B	0.101 (2)	0.0716 (19)	0.0727 (19)	−0.0136 (17)	−0.0279 (17)	0.0330 (15)
C1A	0.0312 (10)	0.0281 (9)	0.0380 (10)	0.0034 (7)	−0.0103 (8)	0.0037 (8)
O12A	0.0312 (7)	0.0457 (8)	0.0549 (9)	0.0008 (6)	−0.0157 (6)	0.0150 (7)
O9A	0.0504 (9)	0.0500 (9)	0.0365 (8)	0.0156 (7)	−0.0163 (7)	0.0022 (6)
N8A	0.0395 (9)	0.0367 (9)	0.0354 (9)	0.0147 (8)	−0.0066 (7)	0.0016 (7)
C9A	0.0305 (9)	0.0323 (10)	0.0344 (10)	0.0026 (8)	−0.0086 (8)	0.0022 (8)
O13A	0.0475 (9)	0.0455 (9)	0.0692 (11)	0.0064 (7)	−0.0221 (8)	0.0192 (8)
O7A'	0.0623 (10)	0.0355 (8)	0.0500 (9)	0.0118 (7)	−0.0041 (8)	−0.0015 (7)
C11A	0.0325 (10)	0.0314 (10)	0.0390 (11)	0.0029 (8)	−0.0126 (8)	0.0021 (8)
C7A	0.0365 (10)	0.0396 (11)	0.0332 (10)	0.0015 (8)	−0.0074 (8)	−0.0003 (8)
C10A	0.0294 (9)	0.0294 (9)	0.0322 (10)	0.0035 (7)	−0.0095 (8)	0.0001 (7)
N14A	0.0348 (9)	0.0306 (8)	0.0509 (10)	−0.0021 (7)	−0.0107 (8)	0.0028 (7)
C1A'	0.0311 (10)	0.0357 (10)	0.0417 (11)	−0.0013 (8)	−0.0089 (8)	0.0011 (8)
C2A	0.0347 (10)	0.0389 (11)	0.0337 (10)	−0.0007 (8)	−0.0106 (8)	0.0039 (8)
C16A	0.0434 (11)	0.0382 (11)	0.0385 (11)	0.0031 (9)	−0.0106 (9)	−0.0069 (9)
C13A	0.0367 (10)	0.0343 (10)	0.0442 (11)	0.0033 (8)	−0.0141 (9)	0.0051 (9)
C2A'	0.0384 (10)	0.0316 (10)	0.0383 (11)	−0.0006 (8)	−0.0090 (9)	−0.0039 (8)
C3A'	0.0532 (13)	0.0374 (11)	0.0450 (12)	−0.0072 (10)	−0.0099 (10)	0.0053 (9)
C3A	0.0544 (13)	0.0573 (14)	0.0417 (12)	0.0022 (11)	−0.0171 (11)	0.0096 (10)
C15A	0.0451 (12)	0.0357 (11)	0.0536 (13)	0.0020 (9)	−0.0117 (10)	−0.0092 (9)
C6A	0.0553 (14)	0.0508 (13)	0.0428 (13)	0.0082 (11)	−0.0045 (10)	−0.0073 (10)
O9A'	0.1098 (16)	0.0449 (10)	0.0646 (12)	0.0027 (10)	0.0074 (11)	0.0184 (9)
C6A'	0.0436 (12)	0.0444 (13)	0.0654 (15)	0.0104 (10)	0.0025 (11)	0.0081 (11)
C4A'	0.0549 (14)	0.0604 (15)	0.0526 (14)	−0.0031 (12)	0.0070 (12)	0.0084 (12)
C4A	0.0676 (16)	0.0842 (19)	0.0338 (12)	−0.0039 (14)	−0.0156 (11)	0.0072 (12)
C17A	0.0480 (13)	0.0396 (12)	0.0730 (16)	−0.0069 (10)	−0.0091 (12)	0.0135 (11)
C5A	0.0637 (16)	0.0794 (18)	0.0354 (12)	−0.0022 (14)	−0.0040 (11)	−0.0119 (12)
C8A'	0.0543 (14)	0.0403 (13)	0.090 (2)	0.0068 (11)	−0.0166 (14)	0.0033 (12)
C5A'	0.0538 (15)	0.0607 (16)	0.0698 (17)	0.0129 (12)	0.0144 (13)	0.0020 (13)
N8	0.0456 (10)	0.0458 (10)	0.0310 (9)	−0.0206 (8)	−0.0067 (7)	0.0022 (7)
O12	0.0506 (9)	0.0326 (7)	0.0507 (9)	−0.0067 (6)	0.0046 (7)	0.0040 (6)
C1	0.0315 (10)	0.0352 (10)	0.0326 (10)	−0.0048 (8)	−0.0066 (8)	0.0031 (8)
C2	0.0374 (10)	0.0339 (10)	0.0307 (10)	−0.0024 (8)	−0.0046 (8)	0.0006 (8)
C7	0.0418 (11)	0.0346 (10)	0.0305 (10)	−0.0045 (8)	−0.0071 (8)	0.0015 (8)
O9	0.0753 (11)	0.0630 (10)	0.0348 (8)	−0.0380 (9)	−0.0126 (8)	0.0138 (7)
O13	0.0449 (9)	0.0522 (9)	0.0619 (10)	−0.0192 (7)	−0.0023 (8)	0.0087 (8)
C10	0.0333 (10)	0.0345 (10)	0.0293 (9)	−0.0060 (8)	−0.0088 (8)	0.0019 (8)
C11	0.0358 (10)	0.0372 (11)	0.0365 (11)	−0.0057 (8)	−0.0049 (8)	0.0047 (8)
N14	0.0338 (9)	0.0466 (10)	0.0458 (10)	0.0019 (7)	−0.0091 (8)	0.0045 (8)
C9	0.0406 (11)	0.0379 (11)	0.0318 (10)	−0.0099 (9)	−0.0070 (9)	0.0035 (8)
C13	0.0380 (11)	0.0403 (11)	0.0372 (11)	−0.0088 (9)	−0.0081 (9)	0.0046 (9)
C1'	0.0447 (12)	0.0347 (11)	0.0440 (12)	−0.0009 (9)	−0.0041 (9)	0.0012 (9)
C3	0.0424 (11)	0.0434 (12)	0.0374 (11)	−0.0054 (9)	−0.0021 (9)	0.0021 (9)
C4	0.0589 (14)	0.0477 (12)	0.0298 (11)	−0.0007 (10)	−0.0028 (10)	0.0024 (9)
C2'	0.0395 (11)	0.0517 (12)	0.0386 (11)	0.0032 (9)	−0.0106 (9)	−0.0002 (9)
C16	0.0473 (12)	0.0494 (12)	0.0385 (11)	−0.0036 (10)	−0.0184 (10)	0.0034 (9)
C6	0.0535 (13)	0.0474 (12)	0.0387 (12)	−0.0120 (10)	−0.0137 (10)	−0.0025 (9)
C5	0.0679 (15)	0.0490 (13)	0.0326 (11)	−0.0033 (11)	−0.0167 (10)	−0.0033 (9)
O7'	0.0427 (9)	0.153 (2)	0.0628 (12)	−0.0300 (11)	−0.0035 (8)	−0.0395 (12)
C15	0.0451 (12)	0.0608 (14)	0.0531 (14)	−0.0037 (11)	−0.0234 (11)	0.0089 (11)
C3'	0.0448 (12)	0.0591 (14)	0.0462 (13)	0.0061 (11)	−0.0026 (10)	0.0012 (11)
C6'	0.0755 (19)	0.0697 (18)	0.081 (2)	−0.0368 (15)	0.0209 (15)	−0.0341 (15)
O9'	0.0486 (11)	0.178 (2)	0.0603 (12)	−0.0212 (13)	0.0080 (9)	−0.0357 (14)
C17	0.0378 (12)	0.0619 (15)	0.0693 (16)	0.0054 (11)	−0.0058 (11)	0.0029 (12)
C5'	0.113 (3)	0.090 (2)	0.098 (2)	−0.056 (2)	0.034 (2)	−0.056 (2)
C4'	0.087 (2)	0.0551 (16)	0.0642 (17)	−0.0073 (14)	0.0179 (15)	−0.0229 (13)
C8'	0.0470 (14)	0.0657 (17)	0.104 (2)	−0.0104 (12)	−0.0114 (14)	−0.0082 (16)
C10'	0.0645 (18)	0.103 (2)	0.0633 (18)	−0.0090 (16)	0.0159 (14)	−0.0132 (16)

### Geometric parameters (Å, º)

**Table:**

C10B—O9A'	1.392 (3)	N8—C9	1.344 (2)
C10B—H10A	0.9600	N8—C7	1.403 (2)
C10B—H10B	0.9600	N8—H8	0.89 (3)
C10B—H10C	0.9600	O12—C13	1.338 (2)
C1A—N14A	1.465 (2)	O12—C11	1.457 (2)
C1A—C2A	1.506 (3)	C1—N14	1.454 (2)
C1A—C10A	1.541 (2)	C1—C2	1.513 (3)
C1A—C13A	1.548 (3)	C1—C10	1.535 (3)
O12A—C13A	1.341 (2)	C1—C13	1.541 (3)
O12A—C11A	1.452 (2)	C2—C3	1.388 (3)
O9A—C9A	1.225 (2)	C2—C7	1.389 (3)
N8A—C9A	1.343 (2)	C7—C6	1.387 (3)
N8A—C7A	1.403 (2)	O9—C9	1.221 (2)
N8A—H8A	0.89 (2)	O13—C13	1.197 (2)
C9A—C10A	1.512 (2)	C10—C9	1.518 (2)
O13A—C13A	1.195 (2)	C10—C16	1.539 (3)
O7A'—C2A'	1.358 (2)	C10—C11	1.546 (3)
O7A'—C8A'	1.422 (3)	C11—C1'	1.499 (3)
C11A—C1A'	1.499 (3)	C11—H11	0.9800
C11A—C10A	1.551 (2)	N14—C17	1.455 (3)
C11A—H11A	0.9800	N14—C15	1.463 (3)
C7A—C6A	1.386 (3)	C1'—C6'	1.381 (3)
C7A—C2A	1.390 (3)	C1'—C2'	1.383 (3)
C10A—C16A	1.543 (3)	C3—C4	1.373 (3)
N14A—C15A	1.457 (3)	C3—H3	0.9300
N14A—C17A	1.459 (3)	C4—C5	1.378 (3)
C1A'—C6A'	1.388 (3)	C4—H4	0.9300
C1A'—C2A'	1.388 (3)	C2'—O7'	1.343 (3)
C2A—C3A	1.390 (3)	C2'—C3'	1.398 (3)
C16A—C15A	1.519 (3)	C16—C15	1.521 (3)
C16A—H16A	0.9700	C16—H16C	0.9700
C16A—H16B	0.9700	C16—H16D	0.9700
C2A'—C3A'	1.398 (3)	C6—C5	1.374 (3)
C3A'—O9A'	1.359 (3)	C6—H6	0.9300
C3A'—C4A'	1.374 (3)	C5—H5	0.9300
C3A—C4A	1.377 (3)	O7'—C8'	1.374 (3)
C3A—H3A	0.9300	C15—H15C	0.9700
C15A—H15A	0.9700	C15—H15D	0.9700
C15A—H15B	0.9700	C3'—O9'	1.357 (3)
C6A—C5A	1.375 (3)	C3'—C4'	1.366 (4)
C6A—H6A	0.9300	C6'—C5'	1.369 (4)
C6A'—C5A'	1.372 (3)	C6'—H6'	0.9300
C6A'—H6A'	0.9300	O9'—C10'	1.395 (3)
C4A'—C5A'	1.375 (4)	C17—H17D	0.9600
C4A'—H4A'	0.9300	C17—H17E	0.9600
C4A—C5A	1.370 (4)	C17—H17F	0.9600
C4A—H4A	0.9300	C5'—C4'	1.366 (4)
C17A—H17A	0.9600	C5'—H5'	0.9300
C17A—H17B	0.9600	C4'—H4'	0.9300
C17A—H17C	0.9600	C8'—H8'1	0.9600
C5A—H5A	0.9300	C8'—H8'2	0.9600
C8A'—H8A1	0.9600	C8'—H8'3	0.9600
C8A'—H8A2	0.9600	C10'—H10D	0.9600
C8A'—H8A3	0.9600	C10'—H10E	0.9600
C5A'—H5A'	0.9300	C10'—H10F	0.9600
			
O9A'—C10B—H10A	109.5	C9—N8—C7	125.51 (16)
O9A'—C10B—H10B	109.5	C9—N8—H8	117.3 (16)
H10A—C10B—H10B	109.5	C7—N8—H8	116.9 (16)
O9A'—C10B—H10C	109.5	C13—O12—C11	111.27 (15)
H10A—C10B—H10C	109.5	N14—C1—C2	114.61 (16)
H10B—C10B—H10C	109.5	N14—C1—C10	103.07 (15)
N14A—C1A—C2A	113.64 (15)	C2—C1—C10	113.98 (15)
N14A—C1A—C10A	102.66 (14)	N14—C1—C13	115.71 (15)
C2A—C1A—C10A	114.65 (15)	C2—C1—C13	108.15 (15)
N14A—C1A—C13A	115.07 (15)	C10—C1—C13	100.52 (15)
C2A—C1A—C13A	109.39 (15)	C3—C2—C7	118.31 (18)
C10A—C1A—C13A	100.71 (14)	C3—C2—C1	122.57 (17)
C13A—O12A—C11A	110.64 (14)	C7—C2—C1	119.12 (16)
C9A—N8A—C7A	126.00 (17)	C6—C7—C2	120.67 (18)
C9A—N8A—H8A	117.9 (15)	C6—C7—N8	118.95 (17)
C7A—N8A—H8A	116.0 (15)	C2—C7—N8	120.36 (17)
O9A—C9A—N8A	121.57 (17)	C9—C10—C1	113.04 (15)
O9A—C9A—C10A	121.93 (17)	C9—C10—C16	111.75 (15)
N8A—C9A—C10A	116.47 (16)	C1—C10—C16	102.91 (15)
C2A'—O7A'—C8A'	121.12 (18)	C9—C10—C11	111.71 (15)
O12A—C11A—C1A'	109.37 (15)	C1—C10—C11	102.04 (14)
O12A—C11A—C10A	103.00 (14)	C16—C10—C11	114.72 (16)
C1A'—C11A—C10A	117.58 (15)	O12—C11—C1'	108.71 (15)
O12A—C11A—H11A	108.8	O12—C11—C10	102.87 (15)
C1A'—C11A—H11A	108.8	C1'—C11—C10	117.93 (16)
C10A—C11A—H11A	108.8	O12—C11—H11	109.0
C6A—C7A—C2A	120.61 (18)	C1'—C11—H11	109.0
C6A—C7A—N8A	118.81 (18)	C10—C11—H11	109.0
C2A—C7A—N8A	120.56 (17)	C1—N14—C17	118.80 (17)
C9A—C10A—C1A	114.39 (15)	C1—N14—C15	104.91 (16)
C9A—C10A—C16A	111.90 (15)	C17—N14—C15	113.96 (18)
C1A—C10A—C16A	103.25 (14)	O9—C9—N8	121.40 (17)
C9A—C10A—C11A	110.44 (14)	O9—C9—C10	122.32 (17)
C1A—C10A—C11A	101.78 (14)	N8—C9—C10	116.26 (16)
C16A—C10A—C11A	114.60 (15)	O13—C13—O12	121.44 (19)
C15A—N14A—C17A	113.06 (17)	O13—C13—C1	128.59 (19)
C15A—N14A—C1A	105.39 (15)	O12—C13—C1	109.97 (15)
C17A—N14A—C1A	118.68 (17)	C6'—C1'—C2'	119.2 (2)
C6A'—C1A'—C2A'	119.18 (19)	C6'—C1'—C11	121.6 (2)
C6A'—C1A'—C11A	121.77 (18)	C2'—C1'—C11	119.20 (18)
C2A'—C1A'—C11A	119.05 (17)	C4—C3—C2	121.04 (19)
C7A—C2A—C3A	118.03 (19)	C4—C3—H3	119.5
C7A—C2A—C1A	119.21 (16)	C2—C3—H3	119.5
C3A—C2A—C1A	122.74 (18)	C3—C4—C5	120.03 (19)
C15A—C16A—C10A	105.86 (16)	C3—C4—H4	120.0
C15A—C16A—H16A	110.6	C5—C4—H4	120.0
C10A—C16A—H16A	110.6	O7'—C2'—C1'	113.90 (19)
C15A—C16A—H16B	110.6	O7'—C2'—C3'	125.9 (2)
C10A—C16A—H16B	110.6	C1'—C2'—C3'	120.1 (2)
H16A—C16A—H16B	108.7	C15—C16—C10	105.64 (16)
O13A—C13A—O12A	121.52 (18)	C15—C16—H16C	110.6
O13A—C13A—C1A	128.10 (18)	C10—C16—H16C	110.6
O12A—C13A—C1A	110.37 (15)	C15—C16—H16D	110.6
O7A'—C2A'—C1A'	115.13 (17)	C10—C16—H16D	110.6
O7A'—C2A'—C3A'	124.65 (18)	H16C—C16—H16D	108.7
C1A'—C2A'—C3A'	119.92 (19)	C5—C6—C7	119.8 (2)
O9A'—C3A'—C4A'	122.8 (2)	C5—C6—H6	120.1
O9A'—C3A'—C2A'	117.4 (2)	C7—C6—H6	120.1
C4A'—C3A'—C2A'	119.8 (2)	C6—C5—C4	120.1 (2)
C4A—C3A—C2A	121.3 (2)	C6—C5—H5	120.0
C4A—C3A—H3A	119.4	C4—C5—H5	120.0
C2A—C3A—H3A	119.4	C2'—O7'—C8'	126.5 (2)
N14A—C15A—C16A	104.53 (16)	N14—C15—C16	104.96 (16)
N14A—C15A—H15A	110.8	N14—C15—H15C	110.8
C16A—C15A—H15A	110.8	C16—C15—H15C	110.8
N14A—C15A—H15B	110.8	N14—C15—H15D	110.8
C16A—C15A—H15B	110.8	C16—C15—H15D	110.8
H15A—C15A—H15B	108.9	H15C—C15—H15D	108.8
C5A—C6A—C7A	119.9 (2)	O9'—C3'—C4'	122.5 (2)
C5A—C6A—H6A	120.0	O9'—C3'—C2'	118.2 (2)
C7A—C6A—H6A	120.0	C4'—C3'—C2'	119.4 (2)
C3A'—O9A'—C10B	119.3 (2)	C5'—C6'—C1'	120.0 (2)
C5A'—C6A'—C1A'	120.4 (2)	C5'—C6'—H6'	120.0
C5A'—C6A'—H6A'	119.8	C1'—C6'—H6'	120.0
C1A'—C6A'—H6A'	119.8	C3'—O9'—C10'	119.0 (2)
C3A'—C4A'—C5A'	120.2 (2)	N14—C17—H17D	109.5
C3A'—C4A'—H4A'	119.9	N14—C17—H17E	109.5
C5A'—C4A'—H4A'	119.9	H17D—C17—H17E	109.5
C5A—C4A—C3A	119.8 (2)	N14—C17—H17F	109.5
C5A—C4A—H4A	120.1	H17D—C17—H17F	109.5
C3A—C4A—H4A	120.1	H17E—C17—H17F	109.5
N14A—C17A—H17A	109.5	C4'—C5'—C6'	120.9 (3)
N14A—C17A—H17B	109.5	C4'—C5'—H5'	119.6
H17A—C17A—H17B	109.5	C6'—C5'—H5'	119.6
N14A—C17A—H17C	109.5	C3'—C4'—C5'	120.3 (2)
H17A—C17A—H17C	109.5	C3'—C4'—H4'	119.8
H17B—C17A—H17C	109.5	C5'—C4'—H4'	119.8
C4A—C5A—C6A	120.3 (2)	O7'—C8'—H8'1	109.5
C4A—C5A—H5A	119.8	O7'—C8'—H8'2	109.5
C6A—C5A—H5A	119.8	H8'1—C8'—H8'2	109.5
O7A'—C8A'—H8A1	109.5	O7'—C8'—H8'3	109.5
O7A'—C8A'—H8A2	109.5	H8'1—C8'—H8'3	109.5
H8A1—C8A'—H8A2	109.5	H8'2—C8'—H8'3	109.5
O7A'—C8A'—H8A3	109.5	O9'—C10'—H10D	109.5
H8A1—C8A'—H8A3	109.5	O9'—C10'—H10E	109.5
H8A2—C8A'—H8A3	109.5	H10D—C10'—H10E	109.5
C6A'—C5A'—C4A'	120.5 (2)	O9'—C10'—H10F	109.5
C6A'—C5A'—H5A'	119.7	H10D—C10'—H10F	109.5
C4A'—C5A'—H5A'	119.7	H10E—C10'—H10F	109.5
			
C7A—N8A—C9A—O9A	169.07 (18)	N14—C1—C2—C3	−82.4 (2)
C7A—N8A—C9A—C10A	−12.8 (3)	C10—C1—C2—C3	159.17 (18)
C13A—O12A—C11A—C1A'	−153.09 (16)	C13—C1—C2—C3	48.3 (2)
C13A—O12A—C11A—C10A	−27.3 (2)	N14—C1—C2—C7	98.4 (2)
C9A—N8A—C7A—C6A	177.86 (19)	C10—C1—C2—C7	−20.0 (3)
C9A—N8A—C7A—C2A	−4.0 (3)	C13—C1—C2—C7	−130.91 (18)
O9A—C9A—C10A—C1A	−150.95 (18)	C3—C2—C7—C6	0.1 (3)
N8A—C9A—C10A—C1A	30.9 (2)	C1—C2—C7—C6	179.38 (19)
O9A—C9A—C10A—C16A	−34.0 (2)	C3—C2—C7—N8	178.38 (18)
N8A—C9A—C10A—C16A	147.90 (17)	C1—C2—C7—N8	−2.4 (3)
O9A—C9A—C10A—C11A	95.0 (2)	C9—N8—C7—C6	−175.7 (2)
N8A—C9A—C10A—C11A	−83.2 (2)	C9—N8—C7—C2	6.0 (3)
N14A—C1A—C10A—C9A	90.48 (17)	N14—C1—C10—C9	−86.85 (18)
C2A—C1A—C10A—C9A	−33.2 (2)	C2—C1—C10—C9	38.0 (2)
C13A—C1A—C10A—C9A	−150.54 (15)	C13—C1—C10—C9	153.41 (16)
N14A—C1A—C10A—C16A	−31.36 (17)	N14—C1—C10—C16	33.86 (18)
C2A—C1A—C10A—C16A	−155.09 (15)	C2—C1—C10—C16	158.69 (16)
C13A—C1A—C10A—C16A	87.62 (16)	C13—C1—C10—C16	−85.88 (17)
N14A—C1A—C10A—C11A	−150.43 (14)	N14—C1—C10—C11	153.04 (14)
C2A—C1A—C10A—C11A	85.84 (17)	C2—C1—C10—C11	−82.13 (18)
C13A—C1A—C10A—C11A	−31.45 (17)	C13—C1—C10—C11	33.30 (17)
O12A—C11A—C10A—C9A	158.13 (15)	C13—O12—C11—C1'	148.84 (17)
C1A'—C11A—C10A—C9A	−81.6 (2)	C13—O12—C11—C10	23.1 (2)
O12A—C11A—C10A—C1A	36.27 (17)	C9—C10—C11—O12	−156.02 (15)
C1A'—C11A—C10A—C1A	156.57 (16)	C1—C10—C11—O12	−34.98 (17)
O12A—C11A—C10A—C16A	−74.39 (18)	C16—C10—C11—O12	75.49 (19)
C1A'—C11A—C10A—C16A	45.9 (2)	C9—C10—C11—C1'	84.4 (2)
C2A—C1A—N14A—C15A	168.15 (16)	C1—C10—C11—C1'	−154.55 (16)
C10A—C1A—N14A—C15A	43.74 (18)	C16—C10—C11—C1'	−44.1 (2)
C13A—C1A—N14A—C15A	−64.6 (2)	C2—C1—N14—C17	62.7 (2)
C2A—C1A—N14A—C17A	−64.0 (2)	C10—C1—N14—C17	−172.91 (18)
C10A—C1A—N14A—C17A	171.59 (17)	C13—C1—N14—C17	−64.3 (2)
C13A—C1A—N14A—C17A	63.2 (2)	C2—C1—N14—C15	−168.57 (16)
O12A—C11A—C1A'—C6A'	26.3 (3)	C10—C1—N14—C15	−44.15 (18)
C10A—C11A—C1A'—C6A'	−90.6 (2)	C13—C1—N14—C15	64.5 (2)
O12A—C11A—C1A'—C2A'	−153.38 (17)	C7—N8—C9—O9	−167.2 (2)
C10A—C11A—C1A'—C2A'	89.7 (2)	C7—N8—C9—C10	14.3 (3)
C6A—C7A—C2A—C3A	0.3 (3)	C1—C10—C9—O9	145.7 (2)
N8A—C7A—C2A—C3A	−177.79 (19)	C16—C10—C9—O9	30.2 (3)
C6A—C7A—C2A—C1A	178.77 (18)	C11—C10—C9—O9	−99.9 (2)
N8A—C7A—C2A—C1A	0.6 (3)	C1—C10—C9—N8	−35.7 (2)
N14A—C1A—C2A—C7A	−99.4 (2)	C16—C10—C9—N8	−151.28 (19)
C10A—C1A—C2A—C7A	18.2 (2)	C11—C10—C9—N8	78.7 (2)
C13A—C1A—C2A—C7A	130.44 (18)	C11—O12—C13—O13	179.06 (19)
N14A—C1A—C2A—C3A	78.9 (2)	C11—O12—C13—C1	−1.1 (2)
C10A—C1A—C2A—C3A	−163.43 (18)	N14—C1—C13—O13	48.4 (3)
C13A—C1A—C2A—C3A	−51.2 (2)	C2—C1—C13—O13	−81.7 (3)
C9A—C10A—C16A—C15A	−114.59 (18)	C10—C1—C13—O13	158.5 (2)
C1A—C10A—C16A—C15A	8.91 (19)	N14—C1—C13—O12	−131.46 (18)
C11A—C10A—C16A—C15A	118.69 (17)	C2—C1—C13—O12	98.43 (18)
C11A—O12A—C13A—O13A	−174.39 (19)	C10—C1—C13—O12	−21.30 (19)
C11A—O12A—C13A—C1A	6.6 (2)	O12—C11—C1'—C6'	−19.7 (3)
N14A—C1A—C13A—O13A	−52.4 (3)	C10—C11—C1'—C6'	96.8 (3)
C2A—C1A—C13A—O13A	76.9 (3)	O12—C11—C1'—C2'	159.99 (18)
C10A—C1A—C13A—O13A	−162.0 (2)	C10—C11—C1'—C2'	−83.5 (2)
N14A—C1A—C13A—O12A	126.50 (17)	C7—C2—C3—C4	−1.5 (3)
C2A—C1A—C13A—O12A	−104.16 (18)	C1—C2—C3—C4	179.28 (19)
C10A—C1A—C13A—O12A	16.9 (2)	C2—C3—C4—C5	1.9 (3)
C8A'—O7A'—C2A'—C1A'	−146.66 (19)	C6'—C1'—C2'—O7'	175.2 (3)
C8A'—O7A'—C2A'—C3A'	39.6 (3)	C11—C1'—C2'—O7'	−4.5 (3)
C6A'—C1A'—C2A'—O7A'	−175.19 (19)	C6'—C1'—C2'—C3'	−1.2 (4)
C11A—C1A'—C2A'—O7A'	4.5 (3)	C11—C1'—C2'—C3'	179.14 (19)
C6A'—C1A'—C2A'—C3A'	−1.1 (3)	C9—C10—C16—C15	109.63 (19)
C11A—C1A'—C2A'—C3A'	178.60 (18)	C1—C10—C16—C15	−12.0 (2)
O7A'—C2A'—C3A'—O9A'	−2.0 (3)	C11—C10—C16—C15	−121.91 (18)
C1A'—C2A'—C3A'—O9A'	−175.4 (2)	C2—C7—C6—C5	0.9 (3)
O7A'—C2A'—C3A'—C4A'	175.2 (2)	N8—C7—C6—C5	−177.4 (2)
C1A'—C2A'—C3A'—C4A'	1.7 (3)	C7—C6—C5—C4	−0.5 (3)
C7A—C2A—C3A—C4A	1.5 (3)	C3—C4—C5—C6	−0.8 (3)
C1A—C2A—C3A—C4A	−176.8 (2)	C1'—C2'—O7'—C8'	164.7 (3)
C17A—N14A—C15A—C16A	−169.27 (17)	C3'—C2'—O7'—C8'	−19.2 (4)
C1A—N14A—C15A—C16A	−38.11 (19)	C1—N14—C15—C16	36.4 (2)
C10A—C16A—C15A—N14A	16.9 (2)	C17—N14—C15—C16	167.98 (18)
C2A—C7A—C6A—C5A	−1.4 (3)	C10—C16—C15—N14	−14.0 (2)
N8A—C7A—C6A—C5A	176.7 (2)	O7'—C2'—C3'—O9'	6.2 (4)
C4A'—C3A'—O9A'—C10B	24.3 (4)	C1'—C2'—C3'—O9'	−177.9 (2)
C2A'—C3A'—O9A'—C10B	−158.7 (2)	O7'—C2'—C3'—C4'	−174.0 (3)
C2A'—C1A'—C6A'—C5A'	−0.1 (4)	C1'—C2'—C3'—C4'	1.9 (4)
C11A—C1A'—C6A'—C5A'	−179.8 (2)	C2'—C1'—C6'—C5'	0.0 (5)
O9A'—C3A'—C4A'—C5A'	175.9 (3)	C11—C1'—C6'—C5'	179.6 (3)
C2A'—C3A'—C4A'—C5A'	−1.1 (4)	C4'—C3'—O9'—C10'	−23.3 (4)
C2A—C3A—C4A—C5A	−2.3 (4)	C2'—C3'—O9'—C10'	156.6 (3)
C3A—C4A—C5A—C6A	1.2 (4)	C1'—C6'—C5'—C4'	0.6 (6)
C7A—C6A—C5A—C4A	0.6 (4)	O9'—C3'—C4'—C5'	178.5 (3)
C1A'—C6A'—C5A'—C4A'	0.7 (4)	C2'—C3'—C4'—C5'	−1.4 (5)
C3A'—C4A'—C5A'—C6A'	−0.1 (4)	C6'—C5'—C4'—C3'	0.1 (6)

### Hydrogen-bond geometry (Å, º)

**Table:**

*D*—H···*A*	*D*—H	H···*A*	*D*···*A*	*D*—H···*A*
N8—H8···O9^i^	0.89 (3)	2.01 (3)	2.903 (2)	177 (2)
N8*A*—H8*A*···O9*A*^ii^	0.89 (2)	2.07 (3)	2.958 (2)	175 (2)
C6*A*′—H6*A*′···O13*A*^iii^	0.93	2.43	3.322 (3)	161

Symmetry codes: (i) −*x*, −*y*+1, −*z*+1; (ii) −*x*+1, −*y*+1, −*z*; (iii) −*x*+2, −*y*, −*z*.
