# Supplementary figures and images for: Crystal structure of 11-(2,3-di­meth­oxy­phen­yl)-14-methyl-12-oxa-8,14-di­aza­tetra­cyclo­[8.3.3.01,10.02,7]hexa­deca-2(7),3,5-triene-9,13-dione
Source: Acta Crystallogr E Crystallogr Commun. 2015 Apr 9;71(Pt 5):o293–4. doi: 10.1107/S2056989015006386 (PMC4420094; doi:10.1107/S2056989015006386)

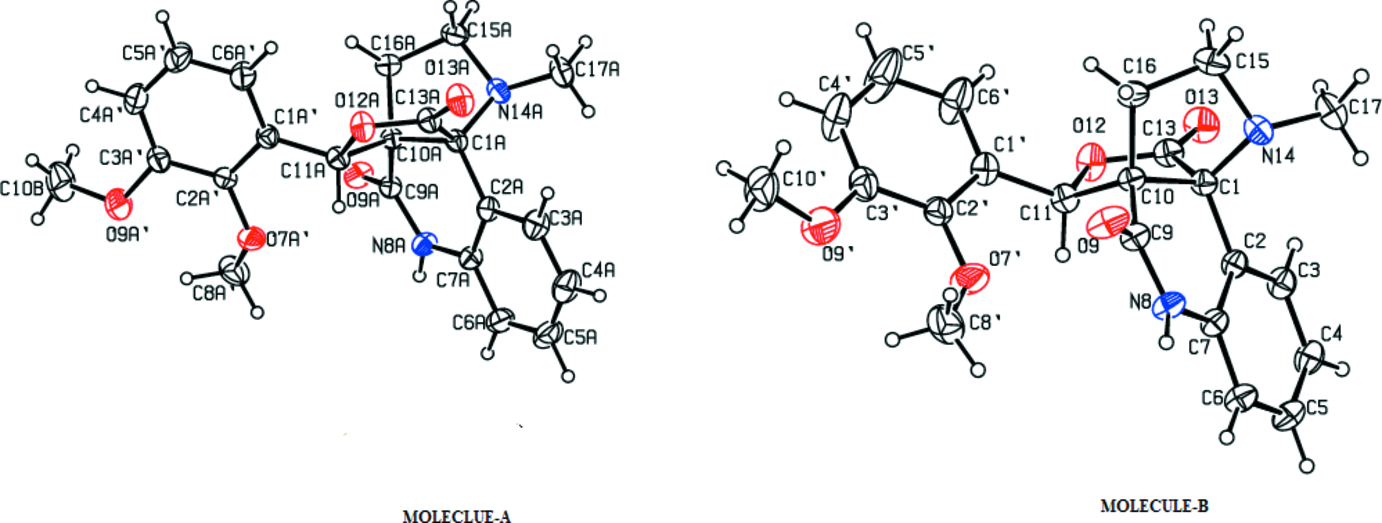

Supplement: Supplementary file 4 [file e-71-0o293-fig1.tif]

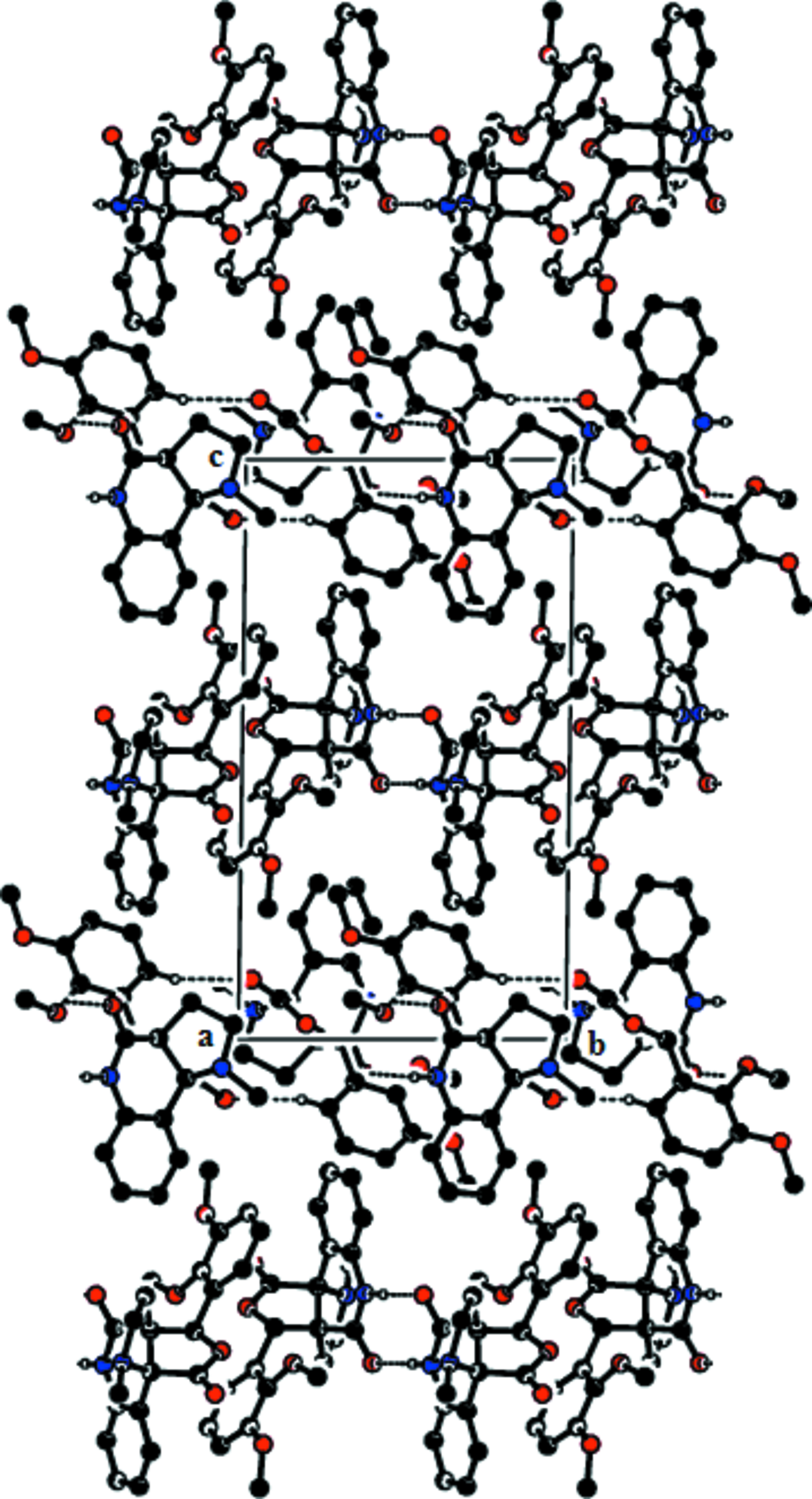

Supplement: Supplementary file 5 [file e-71-0o293-fig2.tif]

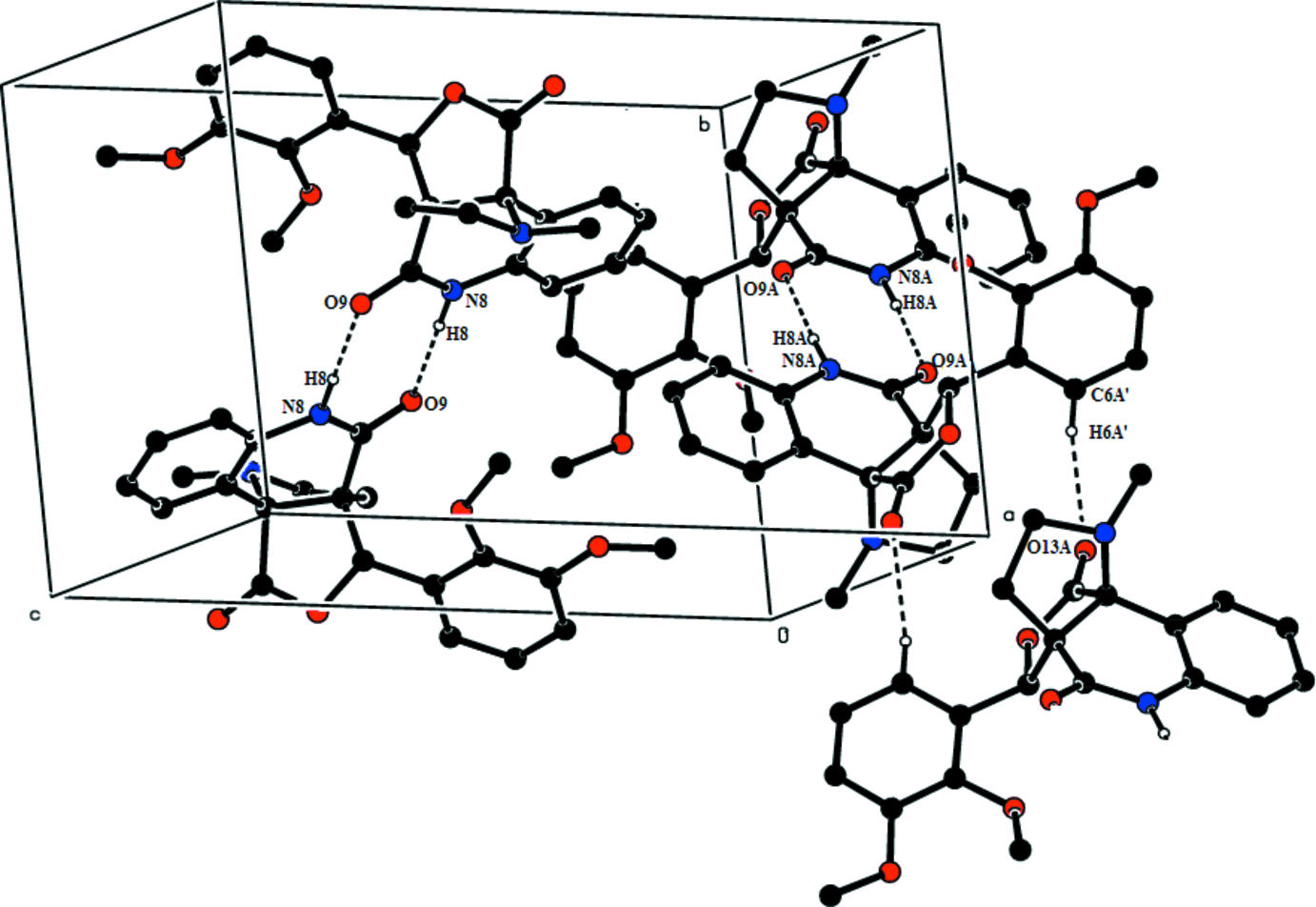

Supplement: Supplementary file 6 [file e-71-0o293-fig3.tif]
